# DPEP1 Balance GSH Involve in Cadmium Stress Response in Blood Clam *Tegillarca granosa*

**DOI:** 10.3389/fphys.2018.00964

**Published:** 2018-07-20

**Authors:** Danli Song, Zhihua Lin, Yongjun Yuan, Guang Qian, Chenghua Li, Yongbo Bao

**Affiliations:** ^1^Zhejiang Key Laboratory of Aquatic Germplasm Resources, College of Biological & Environmental Sciences, Zhejiang Wanli University, Ningbo, China; ^2^School of Marine Sciences, Ningbo University, Ningbo, China

**Keywords:** *Tegillarca granosa*, DPEP1, cadmium, enzyme activity, immunohistochemistry

## Abstract

The blood clam, *Tegillarca granosa*, is a benthic filter feeder with a strong capacity to accumulate and tolerate cadmium (Cd). In our previous study, DPEP1 was shown to be significantly up-regulated under Cd stress based on proteomic analysis. To investigate whether DPEP1 is involved in Cd-induced response, the function of DPEP1 in *T. granosa* was investigated by integrated molecular and protein approaches. Rapid amplification cDNA end (RACE) assay was established to achieve the complete cDNA sequence of DPEP1 from *T. granosa*. The full-length cDNA of DPEP1 was 1811 bp, and it contained a 1359-bp open reading frame (ORF), including a 22-amino acid signal peptide. qRT-PCR analysis revealed that DPEP1 was expressed in all examined tissues with the highest expression in gills. At the same time, we investigated DPEP1 gene expression changes after Cd stress at different time points over 96 h. We found that the expression of DPEP1 increased upon initial Cd stress, then it was inhibited, and finally, it was maintained at a low level. Moreover, recombinant DPEP1 showed that higher glutathione (GSH) hydrolysis activity in the temperature range of 30–40°C, and its maximum activity was at pH = 6. Additionally, the results of immunohistochemistry also confirmed that DPEP1 protein was expressed in all test tissues with the highest expression in gills. In addition, there was a positive correlation between QRT-PCR and immunohistochemistry. These results suggested that DPEP1 is probably involved in Cd-induced response by balancing GSH.

## Introduction

Cadmium (Cd), as a non-essential metal, cannot be degraded or bio-transformed; therefore, it can be easily assimilated, stored, and accumulated, causing a wide range of damage to the organism (Henson and Anderson, 2000; Nasreddine and Parent-Massin, 2002; Cuypers et al., 2010; Hwang et al., 2010). The blood clam *Tegillarca granosa*, which is a commercial benthic bivalve, has a strong ability to accumulate and tolerate Cd. In addition, its Cd accumulation capacity is higher than that of oysters and mussels (Gong et al., 2011). Due to the special accumulation and detoxification abilities of *T. granosa*, many studies have been conducted on its immune response after Cd stress in recent decades. A study found that the accumulation of Cd in *T. granosa* was highest in the mantle membrane and that it increased with the duration of exposure (Wang et al., 2013). The 96-h short-term lethal concentration (LC_50_) of Cd for *T. granosa* is approximately 6 mg/L, and Cd can cause gonad, foot, mantle membrane-specific organ lesions (Liu et al., 2012; Wang et al., 2014). In addition, findings suggest that some miRNAs and their target genes and pathways may play critical roles in the response to Cd stress (Bao et al., 2014). At the same time, many cadmium-related genes and their response to Cd stress in *T. granosa* have been studied (Zhou et al., 2013; Li et al., 2015; Cheng et al., 2016; Chen et al., 2017; Qian et al., 2017). Despite many studies on Cd-induced toxicological effects, the mechanisms of Cd-induced reaction are still poorly understood.

Dipeptidase 1 (DPEP1), also known as dehydropeptidase-I, microsomal dipeptidase (MDP) or renal dipeptidase, is a zinc-dependent metalloproteinase that has been shown to process antibiotics, hydrolyze a variety of peptides, including glutathione (GSH) and its conjugates, and participate in leukotriene metabolism (Kozak and Tate, 1982; Nakagawa et al., 1992). Recently, research on DPEP1 has mainly focused on its impact on cancer. Studies have shown that DPEP1 affects the invasion of cancer cells, and because the expression of DPEP1 decreases with pathological differentiation, it can be used as a genetic marker for the treatment of cancer (Okamoto et al., 2011; Eisenach et al., 2013; Tachibana et al., 2017). A study found that changes in DPEP1 expression can lead to an imbalance of (GSH) (Zhang et al., 2012). GSH is one of the most important antioxidants in cells (Meister, 1994). It plays an important role in maintaining normal redox status and antioxidant defense in the body (Ercal et al., 2001). In addition, toxic metal ions can be excreted by binding to GSH to form complexes (Vethanayagam et al., 1999). It has been found that GSH can be combined with Cd to form a certain complex in detoxification (Peng, 2014). In rats, GSH levels decreased in the liver after exposure to a high concentration of Cd, whereas it increased in liver and kidney after exposure to a low concentration of Cd (Bagchi et al., 1996; Karmakar et al., 1998; Liu et al., 2001). In the fish *Tilapia nilotica*, the content of GSH experienced similar changes to those in rats after Cd stress (Hui et al., 2001). In a teleost hepatoma cell line, the treatment of cells with 10 μM CdCl_2_ led to significant increases in GSH (Schlenk and Rice, 1998). In the mollusk *Anodonta woodiana*, GSH is also sensitive to Cd-induced injury and decreased in gills and liver after high concentration of Cd stress (Yang et al., 2015). It indicated that the change of GSH content is an adaptive regulation of Cd exposure. Thus, DPEP1 may be involved in Cd stress defense by hydrolyzing GSH.

In our previous work, a regulation network of *T. granosa* in response to cadmium toxicity was constructed with both metabolic and proteomic data. In addition, it suggested a potential toxicological pathway for Cd and the discovery of many related functional genes (Bao et al., 2016). Glycolysis, sulfur metabolic process, and Ca^2+^ homeostasis were detected in the study, and these processes were connected with the core network. We found that DPEP1 was the core protein in sulfur metabolic processes and Ca^2+^ homeostasis by iTRAQ proteome analysis. And it was significantly up-regulated after 250 μg/L Cd challenge. According to previous studies, Cd has immunotoxicity to the blood clam and it may enter the tissue through Ca^2+^ channel (Shi et al., 2016, 2018). And it is generally agreed that oxidative stress plays an important role in acute Cd poisoning (Pathak and Khandelwal, 2006), and Cd exposure can alter the antioxidant enzyme activities in blood clam (Peng et al., 2015). In addition, some studies have shown that there is a correlation between sulfate metabolism and Cd (Clemens, 2006). So DPEP1 plays an important role in Cd stress response.

To explore the role of DPEP1 in Cd stress, we first cloned the DPEP1 genes of *T. granosa* and analyzed their expression in different configurations and times after Cd stress by qRT-PCR. Then, recombinant DPEP1 was overexpressed and purified using pET-32a (+) expression vector in *Escherichia coli*, and the activity of purified DPEP1 was assessed. Additionally, the expressions of DPEP1 in various tissues and at different times after Cd stress were examined by immunohistochemistry. This work preliminarily explored the related mechanism of DPEP1 on Cd defense in *T. granosa*, generating important information that can help with our understanding of the mechanism of cadmium detoxification in *T. granosa*.

## Materials and Methods

### Experimental Animals and Sampling

Healthy and consistently sized (average shell length of approximately 30 mm) *T. granosa* were collected from an aquaculture farm (Ningbo, China) and acclimatized in 10 L of aerated natural seawater (28 ± 2% salinity, 25 ± 2°C, and less than 0.08 μg/L Cd) for 7 days. The seawater added with *Chaetoceros* stock was changed daily. During acclimatization and experimentation, there was no death in the clams.

For analysis of the tissue distribution of DPEP1 mRNA, the gill, adductor muscle, blood, viscera, foot, mantle, and gonad of five clams were collected, and each tissue sample was mixed in a 1.5-mL tube. We performed three replicates with three different tissue mixtures, and all samples were flash-frozen in liquid nitrogen and stored at −80°C for RNA extraction and cDNA synthesis.

For the Cd challenge experiment, 260 clams were divided into two groups, and one group was exposed to Cd^2+^ (CdCl_2_) with a final gradient concentration of 25 μg/L. Another group was exposed to 250 μg/L Cd^2+^. After 0, 3, 6, 9, 12, 24, 48, and 96 h of exposure, the blood and gill tissue of *T. granosa* were collected to temporal expression analysis. Each time point takes 15 *T. granosa* and divides the tissues into three groups for mixing. In addition, 0 h served as the control group. We performed three replicates with three different tissue mixtures, and all samples were flash-frozen in liquid nitrogen and stored at −80°C for RNA extraction and cDNA synthesis.

For the immunohistochemistry experiment, *T. granosa* was exposed to 25 μg/L Cd^2+^. After 0, 12, and 96 h of exposure, the gill tissues of three *T. granosa* were randomly sampled. In addition, the adductor muscle, viscera, foot, and mantle of three *T. granosa* at 0 h were collected. All tissues were fixed in 10% neutral buffered formalin, conventional methods of dehydration were applied, and the samples were paraffin-embedded and sliced (Love et al., 2003).

### Cloning and Analysis of the Full-Length cDNA of DPEP1

Total RNA was extracted from *T. granosa* using RNAiso plus (Takara, Japan) according to the manufacturer’s instructions. RNA quality was assessed by electrophoresis on 1% agarose gels and quantified spectrophotometrically with a NanoDrop 2000 UV-Vis Spectrophotometer (Thermo Scientific). The full-length cDNA sequence of DPEP1 was synthesized from high-quality total RNA using a SMARTer^TM^ Rapid amplification cDNA end (RACE) cDNA Amplification Kit (Takara, Japan). The partial cDNA sequence of DPEP1 was extracted from our finished transcriptome data of *T. granosa*, and gene specific primers (**Table 1**) were designed for a 5′ and 3′ RACE experiment. The PCR products were purified using a Gel Extraction Kit (Omega, United States), cloned into the pMD18-T simple vector (Takara, Japan), and transformed into *E. coli* DH5α cells (Takara, Japan) according to the supplied protocols. Then the three positive clones for each product were sequenced at Invitrogen (Shanghai, China).

**Table 1:** Primers and interference sequence information in the present study.

Primer	Sequence (5′–3′)	Used for
DPEP1-3	GGCAAATGCGACAGTTTACCAG	3′RACE
DPEP1-5	GCATTGTGGTGTTCTCTCTCAGGTTG	5′RACE
DPEP1-1	GGCAAATGCGACAGTTTACCAGG	Real-time PCR
DPEP1-2	GCCCAAAACTGTGCTCCAACCA	
Tg-18sRNA	CTTTCAAATGTCTGCCCTATCAACT	Real-time PCR
Tg-18sRNA	TCCCGTATTGTTATTTTTCGTCACT	
DPEP1-F	GGATCCGCTGACAGTTGGAAAAGTGAT	Protein fragment
DPEP1-R	CTCGAGAAAGTTTGTGTTACATGGAAGG	amplification

The cDNA sequence of DPEP1 was determined using the BLAST at the National Center for Biotechnology Information^1^, and the amino acid sequence was deduced using the Expert Protein Analysis System^2^. The molecular mass (MM) and theoretical isoelectric point (pI) of DPEP1 were calculated using the ProtParam tool^3^. The transmembrane region was predicted using the TMHMM Server v. 2.0^4^. Domains were detected by the simple modular architecture research tool program^5^, and multiple alignments analysis of each protein was performed by the Clustal Omega Multiple Alignment program^6^.

### RNA Isolation and Real-Time Quantitative PCR

Total RNA was isolated using the RNAiso Plus (TaKara), and its quality was checked by gel electrophoresis and measured A260/A280 with a NanoDrop 2000 UV-Vis Spectrophotometer (Thermo Scientific). Then total RNA was treated DNase to remove contaminated genomic DNA. Total cDNAs were synthesized with 1000 ng of total RNAs in a final volume of 20 μl using the Prime Script^TM^ RT reagent kit (Takara) according to the manufacturer’s instruction and stored at −20°C before analysis. A real-time quantitative PCR experiment was performed on an ABI 7500 Fast (Thermo Fisher Scientific) with the synthesized total cDNA as a template, and primers specific to DPEP1 were designed using the Primer5 program (**Table 1**). The reactions were performed in a total volume of 20 μL with 10 μL of SYBR Green PCR Master Mix (TaKara), 2 μL of cDNA, 1 μL of each primer (10 μM), and 6 μL of RNase-free water. The cycling conditions were 94°C for 15 min, followed by 40 cycles of 94°C for 15 s, 60°C for 20 s, and 70°C for 25 s. Melting analysis of the amplified products was performed at the end of each PCR cycle to confirm that a single PCR product was produced and detected. The 18S rRNA expression was served as an internal reference under the same conditions with primers shown in **Table 1**. Each sample was analyzed in triplicate. The 2^−ΔΔCT^ method was used to determine the relative expression levels of the measured genes (Livak and Schmittgen, 2001). The data were presented as relative mRNA expression levels (means ± SD, *n* = 3).

### Generation of Recombinant DPEP1 *in Vitro*

The coding region of DPEP1 without the transmembrane region was amplified by PCR with specific primers (**Table 1**). The PCR fragment and pET-32a (Novagen, United States) were digested by restriction endonucleases BamH1 and Xhol (NEB, United States) and then ligated them. Then the recombinant plasmid (pET-32a-DPEP1) was transformed into *E. coli* Rosetta (DE3). Positive transformants were incubated in LB medium containing 100 mg/mL ampicillin at 37°C with shaking at 200 rpm. When the culture media reached the OD_600_ = 0.5–0.8, the final concentration of 1.0 mM isopropyl β-D-1-thiogalactopyranoside (IPTG) was added into the LB medium and incubated at 37°C with shaking at 200 rpm for 4 h. After induction, the bacteria were collected by centrifugation at 10000 rpm for 10 min and then resuspended in inclusion washing buffer for sonication treatment. The supernatant and precipitate was obtained by centrifugation at 12000 rpm for 30 min. The precipitate was then re-suspended with inclusion lysate solution and sonicated to collect the supernatant for the second time. The recombinant protein of DPEP1 was purified by a Ni-NTA Sepharose column (Sangon Biotech, Shanghai). The recombinant protein was analyzed by SDS-PAGE to verify purity. The concentration of recombinant protein was quantified by BCA assay using the PierceTM BCA Protein Assay Kit (Thermo scientific, United States) according to the manufacturer’s instructions.

### Enzyme Activity Assay of DPEP1

The enzyme activity assay of recombinant DPEP1 protein was carried out with GSH as the substrate and 5,5′-dithiobis-(2-nitrobenzoic acid) (DTNB) as the chromogenic agent. Stock solutions of DTNB at 4 mM (Solarbio, China) were dissolved in 0.1 mol/L sodium phosphate buffer and prepared 500 μg/mL GSH before using. Measurements were performed by mixing 10 μL of GSH and 10 μL of 1 mg/mL recombinant protein in 96-well plate for 30 min (*n* = 10), then adding 180 μL of DTNB. After brief shaking to mix thoroughly, the mixture was measured continuously for 1 min at room temperature for absorbance at 422 nm using a Spectra Max 190 (Molecular Devices, United States). The optimal temperature for enzyme activity was determined by measuring the activities at temperatures between 4 and 60°C in the chromogenic agent described earlier. The optimal pH was determined by measuring the activities in chromogenic agents at different pH values ranging from pH 2 to pH 10. The 10 μL of GSH with 190 μL of DTNB as a control group and all tests were repeated three times. Making a standard curve based on the reaction of different concentrations of GSH and DTNP. In addition, the enzyme activity of DPEP1 was expressed as a decrease in GSH.

### Immunohistochemistry Analysis of DPEP1

DPEP1 polyclonal antibody was prepared with our recombinant DPEP1. The antiserum was harvested from rabbit (Huabio, China) and stored at −20°C for further experiments. For immunohistochemistry analysis, first the 4 μm sections were deparaffinized (xylene) and dehydrated (graded alcohols). Second, 0.3% methanolic hydrogen peroxide solution was added to the sections, and they were incubated in the dark for 10 min at room temperature and then placed in PBS (pH 7.4) and washed three times for 3 min each. Third, the sections were placed in Boiled EDTA buffer (Huabio, China) (pH 8.0) for microwave repair for 20 min, then cooled at room temperature for 10 min, placed in PBS (pH 7.4) and washed three times for 3 min each. Fourth, 1% BSA (Sigma, United States) was added to the sections in the circle drawn with PAP Pen (Zsgb-Bio, China), and the samples were then incubated for 20 min. Fifth, the sections were incubated with primary antibody (DPEP1 antibody dilution at 1:2000) for 1 h and incubated with secondary antibody using MaxVision^TM^ HRP-Polymer anti-Rabbit IHC Kit (MaxVision, China) for 15 min at room temperature. Sixth, DAB (Gene Tech, China) was added to the sections, and the sections were observed under the microscope for approximately 3 min, and then washed with tap water for color termination. Seventh, the sections were dyed with hematoxylin for 4 min, flushed with tap water, dehydrated with gradient alcohol, and made transparent with xylene. Finally, the sections were sealed with a neutral gum. Tissues that were immunohistochemically stained with PBS instead of primary antibody served as negative controls. The immunohistochemical results were analyzed using Image-Pro Plus software and expressed as density mean.

### Statistical Analysis

Means with SDs were used for descriptive statistics throughout the article (mean ± SD). Data were analyzed and processed by SPSS statistical software. Independent *t*-tests were used to compare with control group. Levels of gene expression in Cd^2+^ challenges were compared using one factor ANOVA. The protein expression is expressed in density mean. Levels of protein expression were also compared using one factors ANOVA. The relationship between density results and biomolecular results was statistically analyzed by Pearson correlation analysis. Any significant differences are indicated with an asterisk at *p* < 0.05 and two asterisks at *p* < 0.01.

## Results

### Analysis of the Full-Length cDNA of DPEP1

**FIGURE 1:**
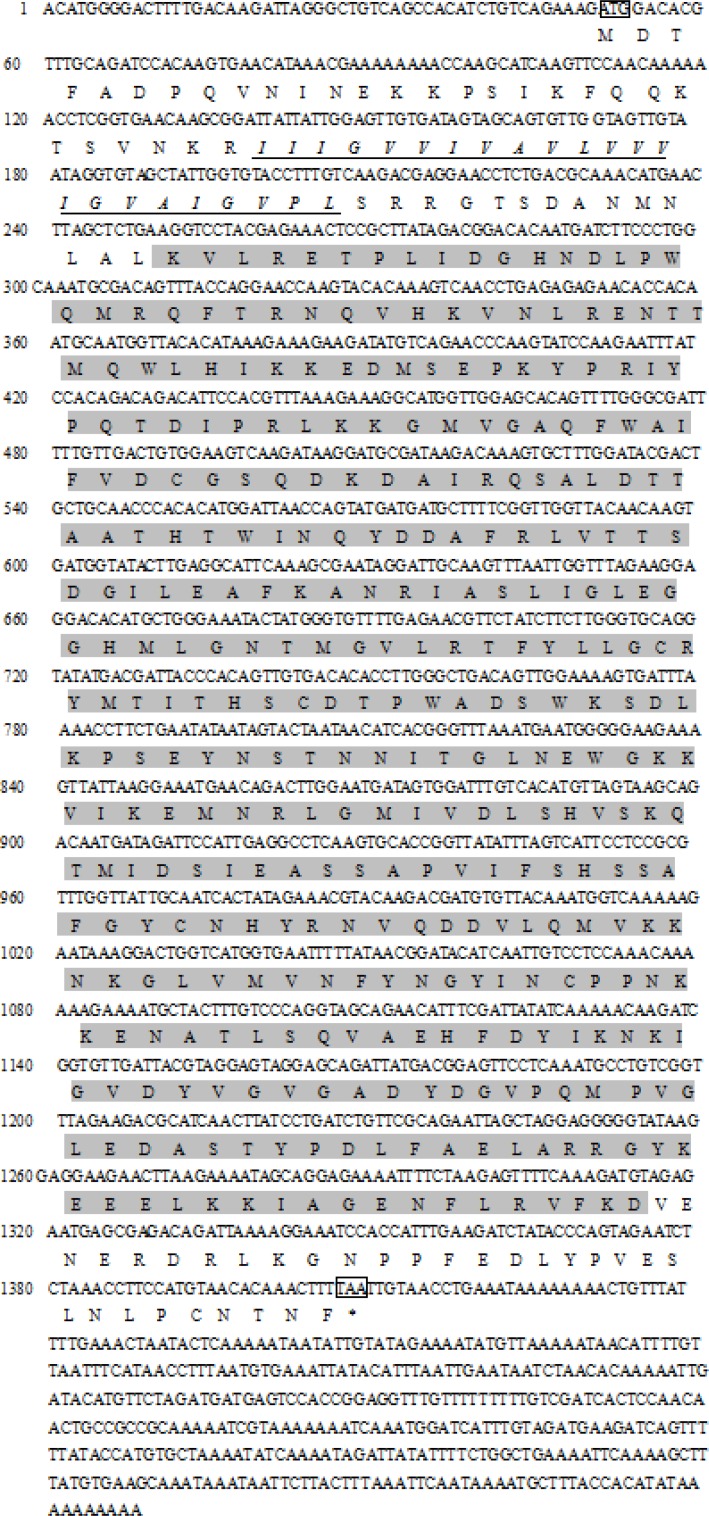
Complete cDNA sequences of DPEP1 from *Tegillarca granosa* and deduced amino acid sequences. The deduced amino acid sequence is shown below the nucleotide sequence in single letter code, and the predicted transmembrane region sequence is indicated with italics and underlined letters. The start and stop codons and tailing signal are framed. The shadowed part belongs to the domain of peptidase family M19.

**FIGURE 2:**
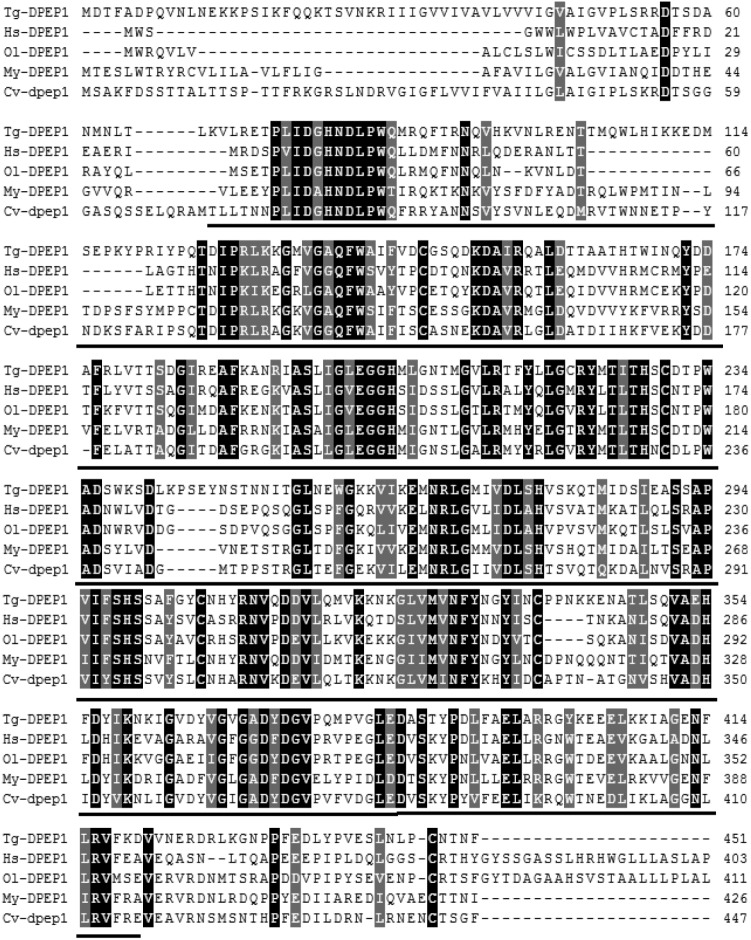
Multiple sequence alignment of DPEP1 from *T. granosa* (Tg-DPEP1) with other known DPEP1 amino acid sequences: *Homo sapiens* (Hs, CAG33685.1), *Oryzias latipes* (Ol, XP_004067177.1), *Mizuhopecten yessoensis* (My, XP_021371422.1), and *Crassostrea virginica* (Cv, XP_022307112.1). Identities are shaded dark, and similarities are shaded gray. The DPEP1 domain regions are underlined.

The full-length cDNA of DPEP1 was 1811 bp, including a 5′UTR of 51 bp, an ORF of 1359 bp encoding 452 amino acid residues and a 3′UTR of 401 bp (**Figure 1**). The predicted MM of the deduced amino acids of DPEP1 was 51 kDa, and the theoretical pI was 7.14. TMHMM prediction revealed that a transmembrane region was located in the 30–52th amino acids of DPEP1. Amino acid sequence analysis indicated that a characteristic feature from residues 68–421 belonged to peptidase family M19. BLAST analysis revealed that DPEP1 shared approximately 45–60% overall identity with counterparts from vertebrates and invertebrates, such as 45% with *Homo sapiens*, 49% with *Oryzias latipes*, 52% with *Mizuhopecten yessoensis*, and 52% with *Crassostrea virginica* (**Figure 2**).

### Tissue Expression of the DPEP1 Gene

**FIGURE 3:**
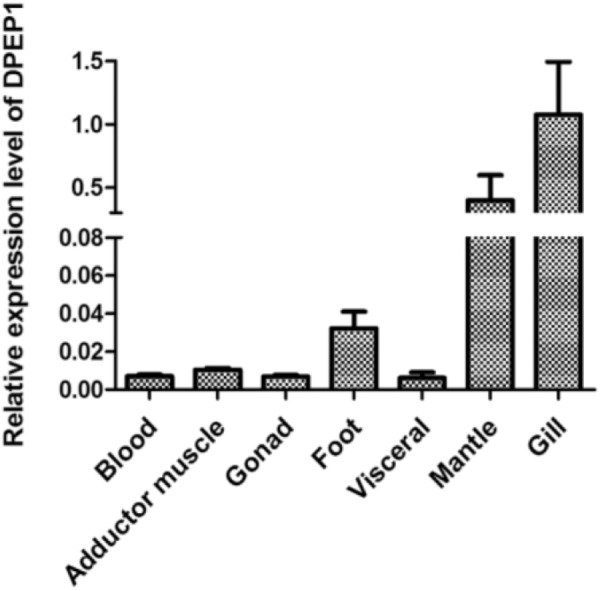
DPEP1 mRNA expression in various tissues. Expression levels were calibrated against blood (control).

The results of DPEP1 spatial distribution are shown in **Figure 3**. The values represent the difference relative to the calibrator tissue (blood). The data show that expression of the mRNA transcript of DPEP1 was detected in all examined tissues, and the mRNA level was most highly expressed in gill and then in mantle, foot, adductor muscle, blood, gonad, and viscera.

### Temporal Expression Analysis of DPEP1 after Cd Challenge

**FIGURE 4:**
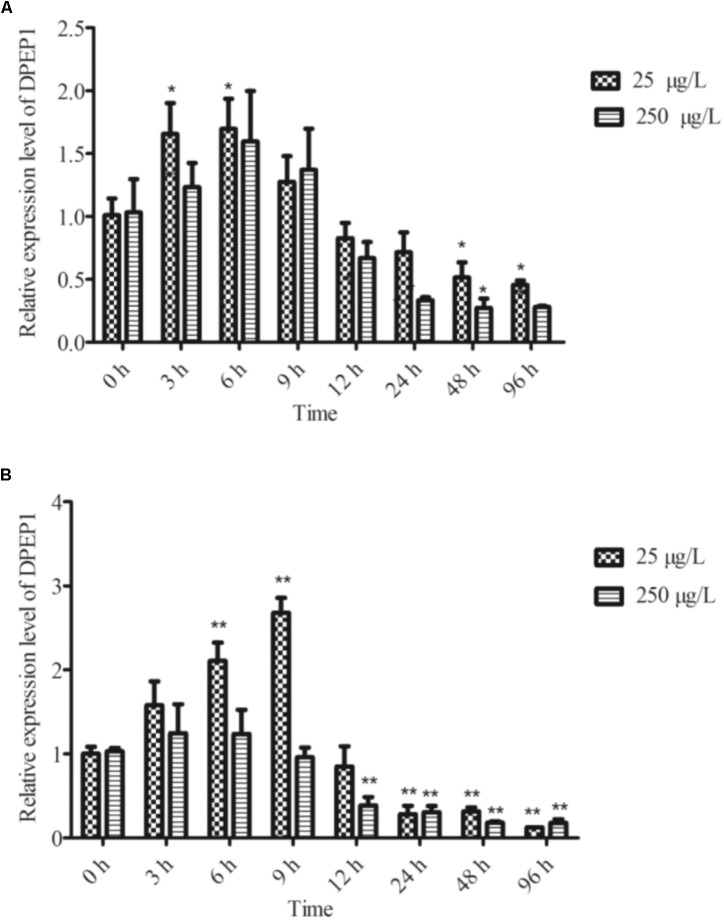
DPEP1 mRNA expression after Cd stress. **(A)** Temporal expression analysis of DPEP1 in gill tissue. **(B)** Temporal expression analysis of DPEP1 in blood. Asterisks indicate significant difference: ^∗^*p* < 0.05 and ^∗∗^*p* < 0.01. Control: samples obtained in 0 h.

The temporal expression of DPEP1 in gills after Cd stress is shown in **Figure 4A**. The expression level of DPEP1 mRNA increased in the first 0–9 h (*p* < 0.05), then it was inhibited from 48 h (*p* < 0.05), and finally, it maintained a low expression level until to 96 h under 25 μg/L Cd^2+^ stress. In addition, it showed no obvious change from 0 to 24 h, then it was inhibited from 48 to 96 h (*p* < 0.05) under 250 μg/L Cd^2+^ stress. The temporal expression of DPEP1 in blood after Cd stress is shown in **Figure 4B**. The expression level of DPEP1 mRNA had no change in the first 0–3 h, then it increased obviously and reached peak expression at 9 h (*p* < 0.01), then it was inhibited from 24 h (*p* < 0.01), and finally, it maintained a low expression level until 96 h under 25 μg/L Cd^2+^ stress. In addition, it showed no obvious change from 0 to 9 h, then it was inhibited from 48 to 96 h (*p* < 0.05) under 250 μg/L Cd^2+^ stress.

### Characterization of the Enzymatic Activity of Recombinant DPEP1

**FIGURE 5:**
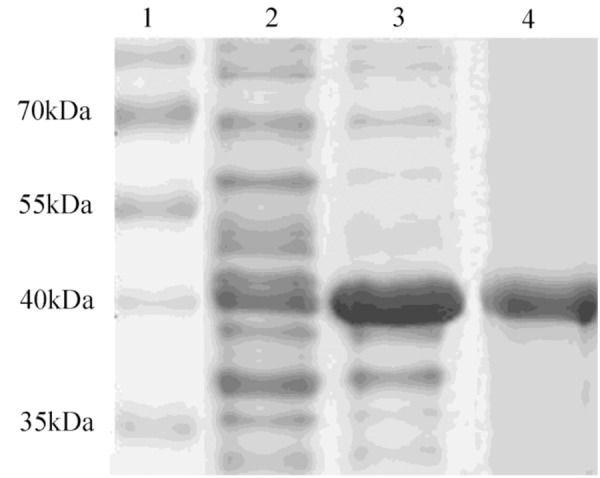
SDS-PAGE analysis of recombinant protein. Lane 1, markers; lane 2, positive transformant *Escherichia coli* without IPTG induction; lane 3, positive transformant *E. coli* induced by IPTG; and lane 4, purified protein.

**FIGURE 6:**
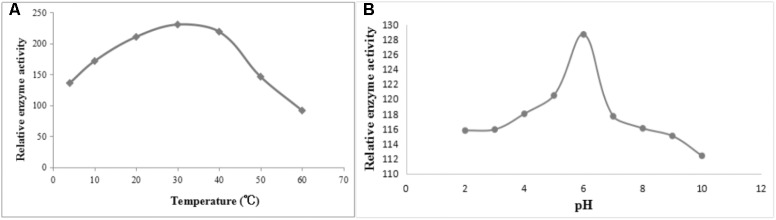
Determination of enzyme activity of recombinant protein. **(A)** Effect of temperature on the enzyme activity of recombinant protein. **(B)** Effect of pH on the enzyme activity of recombinant protein.

After induction with IPTG, the DPEP1 gene was successfully expressed and it could be purified to homogeneity by Hi-Trap Chelating Columns. The result was revealed by SDS-PAGE (**Figure 5**). The MM of the purified product was in good agreement with the predicted MM. The enzyme activities of recombinant DPEP1 was shown by a decrease in GSH. At 25°C, pH = 7.210 ± 13.95 μg (*n* = 10) of GSH can be hydrolyzed in 1 mg of recombinant DPEP1. The purified recombinant DPEP1 displayed that it was influenced by temperature, and maximum activities were at 30∼40°C (**Figure 6A**). In addition, recombinant DPEP1 showed maximum activities at pH = 6 (**Figure 6B**).

### Immunohistochemistry Analysis of DPEP1

**FIGURE 7:**
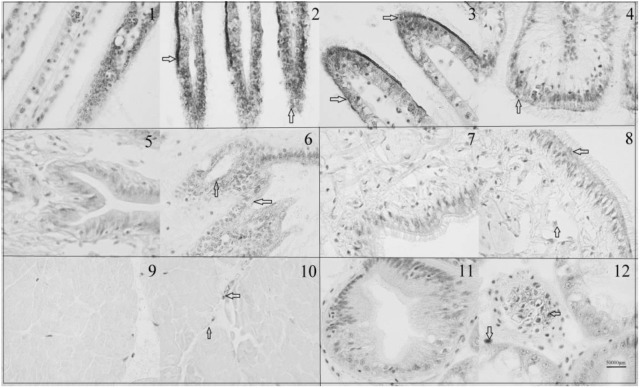
Immunohistochemistry of DPEP1. Tissue was observed under 1000 times microscopes. (1) Negative control of gills at 0 h, (2) gills at 0 h, (3) gills after 12 h of 25 μg/L Cd stress, (4) gills after 96 h of 25 μg/L Cd stress, (5) negative control of mantle, (6) mantle at 0 h, (7) negative control of foot (8) foot at 0 h, (9) negative control of adductor muscle, (10) adductor muscle at 0 h, (11) negative control of viscera, and (12) viscera at 0 h. The DPEP1 protein is expressed in the brown part of the arrowhead. The same organization is marked with a black box.

**FIGURE 8:**
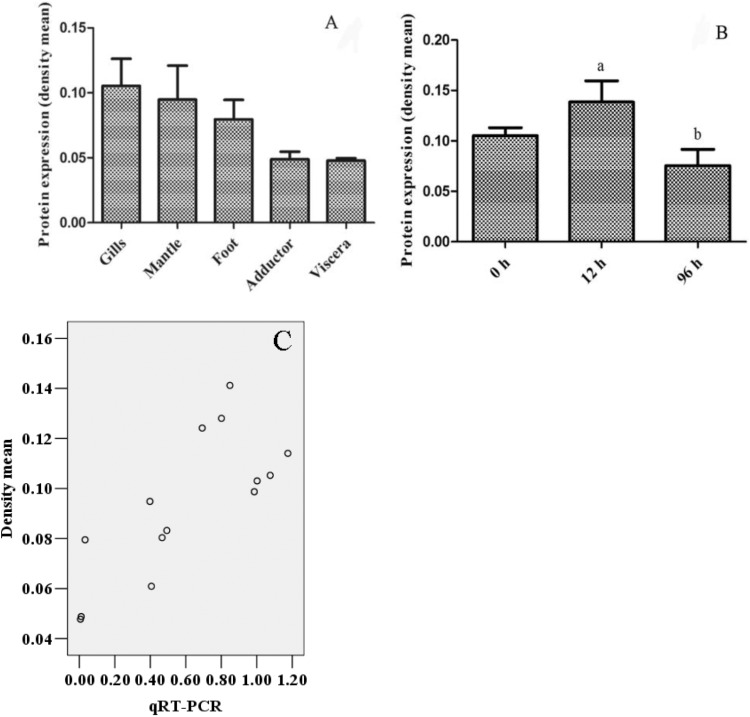
The quantized value of immunohistochemistry (*n* = 3) and a scatter diagram. **(A)** The density mean of various tissues. **(B)** The density mean of gills in different time after 25 μg/L Cd2^+^ stress. The 12 and 96 h are significant difference (*p* < 0.05). **(C)** The scatter diagram of densitometric analysis results with biomolecular analysis results.

The immunohistochemistry of DPEP1 in tissues is shown in **Figure 7**. And the protein expression is shown in **Figures 8A,B**. Immunohistochemistry analysis reviewed that the expression level of DPEP1 protein was the highest in gill and then decreased in mantle, foot, adductor muscle, and viscera. DPEP1 was mainly expressed in epithelial cells at the periphery of the tissue. And it also expressed in some connective tissue and blood sinuses of mantle and viscera. In addition, the expression of DPEP1 in gills was increased at first 12 h then inhibited until 96 h (*p* < 0.05) after 25 μg/L Cd^2+^ stress. Pearson correlation analysis was used to evaluate the relationship between qRT-PCR and immunohistochemistry. There is a linear relationship between the two variables (**Figure 8C**). According to the Shapiro–Wilk test, it matches the normal distribution (*p* > 0.05), and there is no outlier. There was a strong positive correlation between qRT-PCR and immunohistochemistry, *r* = 0.767, *p* < 0.001. The expression trend of DPEP1 at protein level is consistent with the molecular level.

## Discussion

Bivalves have a sedentary and filter-feeding living style, which facilitates the accumulation of pollutants at a high level. Their metal concentration accumulation and integration capabilities and special detoxification capacity has drawn researcher’s attention (Engel and Brouwer, 1984; Bebianno and Serafim, 1998; Gao and Wang, 2017). Studies have shown that of the five most common mollusks along the Zhejiang coast in China, *T. granosa* had the greatest aptitude for the bioaccumulation of Cd (Huang et al., 2007). However, there is a lack of the mechanism study of *T. granosa* in heavy metal toxicity. In *T. granosa*, DPEP1 was significantly up-regulated after 250 μg/L Cd challenge based on iTRAQ proteome analysis in our previous study (Bao et al., 2016), which suggests that DPEP1 might be involved in Cd-induced responses.

In this study, the full-length cDNA of *T. granosa* DPEP1 was cloned for the first time. Amino acid sequence analysis indicated that DPEP1 possessed a structural domain belonging to peptidase family M19 (membrane dipeptidase family, clan MJ) and a hydrophobic region at the carboxylic end of its membrane-bound region. These results are consistent with those other reported DPEP1 sequences (Adachi et al., 1993; Nitanai et al., 2002). DPEP1 is a membrane-bound dipeptidase involved in the degradation of surrounding extracellular matrix components (Mciver et al., 2004). Immunohistochemistry showed that the DPEP1 is mainly expressed in the epithelium. These results are similar to those of human DPEP1 (Inamura et al., 1994). This finding also suggests that DPEP1 may be responsible for the degradation of tissue basement membranes. Therefore, an increase in DPEP1 protein may allow heavy metals to move into the body more easily.

The enzymatic activity of recombinant DPEP1 showed that it can hydrolyze GSH. These results confirmed that DPEP1 plays important roles in the metabolism of GSH and its conjugates (Hooper et al., 1987; Adachi et al., 1990). GSH is widely distributed and is a primary intracellular antioxidant agent (Kidd, 1997). Its thiol (SH-) group on the cysteine residue can scavenge free radicals (Noctor and Foyer, 1998; Dickinson and Forman, 2002). Cd is unable to generate reactive oxygen species (ROS) directly, but Cd-induced oxidative stress is a common phenomenon observed in multiple studies (Dorta et al., 2003; Belyaeva et al., 2006). Therefore, GSH is one of the most important metabolites when dealing with Cd-induced oxidative stress. Due to the thiol groups in GSH, sulfhydryl reactive metals, such as Cd, can easily bind to them to form Cd–GSH (Ercal et al., 2001; Hansen et al., 2006). Complexation by GSH makes free Cd unavailable for cell metabolism, preventing Cd interaction with critical cellular targets, and blocking the mechanisms leading to Cd-induced oxidative stress (Kamiyama et al., 1995; Rana and Verma, 1996; Waisberg et al., 2003). Therefore, during Cd stress, GSH not only neutralizes ROS but also detoxifies Cd directly (Vairavamurthy et al., 2000; Thévenod, 2003). So the change of GSH content plays an important role in the detoxification of cadmium. In addition, studies have found that oxidative stress is related to the SNPs of GSH metabolism-related genes, including DPEP1 (Bowers et al., 2010). Reduced DPEP1 expression has been found to lead to the dysregulation of GSH homeostasis, which affects the maintenance of an optimal redox state in the cellular microenvironment and protects cells against pathological stress (Klaunig et al., 1998; Zhang et al., 2012). These results show that DPEP1 can affect the oxidative stress of the body through the metabolism of GSH. DPEP1 may involve in cadmium stress response on the body by balancing GSH.

Tissue expression analysis by real-time PCR demonstrated that DPEP1 mRNA was expressed in all studied tissues and that it showed the highest expression level in gills and then the next-highest expression in mantle, and immunohistochemistry analysis presented the same distribution at protein level. Gills are the likely primary entry point of waterborne Cd and the main organ for enriching heavy metals in the short term (Domouhtsidou and Dimitriadis, 2000; Paul-Pont et al., 2010). Gills are important in the modulation and clearance of cadmium (Haberkorn et al., 2010). Various damages to gills have also been reported in the mussel *Mytilus galloprovincialis* Lam after Cd stress (Viarengo et al., 1985; Fernandez et al., 2010). Many studies have also shown that genes related to cadmium detoxification in *T. granosa* have a higher expression level in gills (Bao et al., 2016; Qian et al., 2017). The higher transcriptional expression in gills and mantle suggests that DPEP1 may associate with the response to Cd. Additionally, the universal expression of DPEP1 suggests that it might be involved in various metabolic pathways. Temporal expression analysis by real-time PCR demonstrated that DPEP1 mRNA expression peaked in the initial stage, which was followed by a decline, and finally, inhibition after Cd stress at either 25 or 250 μg/L in two tested tissues. This result is similar to metallothioneins (MT) expression in *Crassostrea hongkongensis* after Cd stress (Xu et al., 2015). In addition, immunohistochemistry analysis in gills after 25 μg/L Cd stress also presented the same trend at the protein level. In addition, after Cd stress, the highest expression difference was in the viscera. GSH is mainly synthesized in the viscera, and its synthesis is fast and sensitive (Kamiyama et al., 1995; Liu et al., 1995). When cadmium enters the body, it is enriched in the liver, resulting in high GSH expression. Therefore, DPEP1 is also increased in liver. From this result, we speculate that during initial exposure, the host was in its active defense state; when its cells were oxidatively challenged, it attempted to maintain a redox balance in the internal environment and GSH synthesis increased, resulting in increased expression of DPEP1. After a period of exposure, in order to keep the higher content of GSH, the expression of DPEP1 decreased, and finally, its expression was inhibited. That is, when cadmium stress occurs, the body produces a large number of ROS; in order to reduce the resulting oxidation loss, GSH content will increase until a dynamic balance is maintained, resulting in changes in the expression of DPEP1. These facts suggest that DPEP1 is involved in cadmium stress response by balancing GSH in *T. granosa*.

In summary, the coding sequence of DPEP1 from *T. granosa* was elucidated for the first time. DPEP1 and its Cd response-related functions in *T. granosa* were investigated by molecular and protein approaches in this study. Our data demonstrate the differential expression of DPEP1 in various organs. In addition, low expression of DPEP1 may protect *T. granosa* after Cd stress. DPEP1 is involved in GSH metabolism and may change cell membrane permeability. The function and mechanism of DPEP1 in Cd stress in *T. granosa* should be further studied.

## Author Contributions

YB conceived and designed the experiments. DS, CL, and YY performed the experiments. DS, ZL, and GQ analyzed the data. DS, CL, and GQ contributed reagents, materials, and analysis tools. YB and DS wrote the paper. All authors reviewed the manuscript.

## Conflict of Interest Statement

The authors declare that the research was conducted in the absence of any commercial or financial relationships that could be construed as a potential conflict of interest.

## Footnotes

1 http://www.ncbi.nlm.nih.gov/blast

2 http://www.expasy.org/

3 http://www.expasy.ch/tools/protparam.html

4 http://www.cbs.dtu.dk/services/TMHMM/

5 http://smart.embl-heidelberg.de/

6 http://www.ebi.ac.uk/clustalw/
